# Heterogeneity of Cellular Senescence: Cell Type-Specific and Senescence Stimulus-Dependent Epigenetic Alterations

**DOI:** 10.3390/cells12060927

**Published:** 2023-03-17

**Authors:** Katarzyna Malgorzata Kwiatkowska, Eleni Mavrogonatou, Adamantia Papadopoulou, Claudia Sala, Luciano Calzari, Davide Gentilini, Maria Giulia Bacalini, Daniele Dall’Olio, Gastone Castellani, Francesco Ravaioli, Claudio Franceschi, Paolo Garagnani, Chiara Pirazzini, Dimitris Kletsas

**Affiliations:** 1 Department of Medical and Surgical Sciences (DIMEC), University of Bologna, 40126 Bologna, Italy; 2 Laboratory of Cell Proliferation and Ageing, Institute of Biosciences and Applications, National Centre for Scientific Research “Demokritos”, 15341 Athens, Greece; 3 Bioinformatics and Statistical Genomics Unit, Istituto Auxologico Italiano IRCCS, 20095 Milan, Italy; 4 Department of Brain and Behavioral Sciences, Università di Pavia, 27100 Pavia, Italy; 5 IRCCS Istituto delle Scienze Neurologiche di Bologna, 40139 Bologna, Italy; 6 Laboratory of Systems Medicine of Healthy Aging, Institute of Biology and Biomedicine and Institute of Information Technology, Mathematics and Mechanics, Department of Applied Mathematics, N. I. Lobachevsky State University, 603022 Nizhny Novgorod, Russia; 7 IRCCS Azienda Ospedaliero-Universitaria di Bologna, 40138 Bologna, Italy

**Keywords:** replicative senescence, stress-induced premature senescence, methylation, epigenetics, human fibroblasts, mesenchymal stem cells

## Abstract

The aim of the present study was to provide a comprehensive characterization of whole genome DNA methylation patterns in replicative and ionizing irradiation- or doxorubicin-induced premature senescence, exhaustively exploring epigenetic modifications in three different human cell types: in somatic diploid skin fibroblasts and in bone marrow- and adipose-derived mesenchymal stem cells. With CpG-wise differential analysis, three epigenetic signatures were identified: (a) cell type- and treatment-specific signature; (b) cell type-specific senescence-related signature; and (c) cell type-transversal replicative senescence-related signature. Cluster analysis revealed that only replicative senescent cells created a distinct group reflecting notable alterations in the DNA methylation patterns accompanying this cellular state. Replicative senescence-associated epigenetic changes seemed to be of such an extent that they surpassed interpersonal dissimilarities. Enrichment in pathways linked to the nervous system and involved in the neurological functions was shown after pathway analysis of genes involved in the cell type-transversal replicative senescence-related signature. Although DNA methylation clock analysis provided no statistically significant evidence on epigenetic age acceleration related to senescence, a persistent trend of increased biological age in replicative senescent cultures of all three cell types was observed. Overall, this work indicates the heterogeneity of senescent cells depending on the tissue of origin and the type of senescence inducer that could be putatively translated to a distinct impact on tissue homeostasis.

## 1. Introduction

Aging is characterized by an inevitable general deterioration of diverse tissues and organs and an increased risk for the occurrence of several morbidities, including cancer, cardiovascular diseases and musculoskeletal and neurodegenerative disorders. Thus, shedding light on the molecular mechanisms underlying the induction and manifestation of the aging process at all levels—from the cellular to the organismal one—has become a main research focus, aiming at unraveling or developing ways to ameliorate the aged phenotype and promote a healthy longevity.

Among the hallmarks of aging, cellular senescence has been considered to be a major contributor to age-related pathologies [1,2,3,4,5]. Cellular senescence was first described in human embryonic lung fibroblasts as a state of irreversible cessation of proliferation after serial subculturing in vitro [6]. Besides this type of senescence—known as replicative senescence, being the result of telomere shortening [7]—cells may also be driven to another type of senescence after their exposure to sub-cytotoxic genotoxic stresses—the stress-induced premature senescence (SIPS) [8,9,10]. Apart from their inability to proliferate, senescent cells remain metabolically active and share common phenotypic and molecular traits irrespective of their origin, i.e., their enlarged and flattened morphology, increased cytoplasmic and lysosomal content, lipofuscin accumulation, resistance to apoptosis, disturbance of nuclear structure, formation of senescence-associated heterochromatin foci (SAHF) and up-regulation of cyclin-dependent kinase inhibitors, such as p16^INK4A^ [11,12]. The most critical phenotypic characteristic of senescent cells that defines their role in the tissue as either positive or negative, depending on the context, is their secretome—the so-called senescence-associated secretory phenotype (SASP), consisting of proinflammatory cytokines, extracellular matrix (ECM)-degrading enzymes and ECM components [13,14,15]. Through their SASP, senescent cells may accelerate wound healing and tissue repair under normal conditions [16], but on the other hand, when accumulating in particular tissues, they may support degenerative disorders or even cancer progression via the formation of a permissive microenvironment [14,15]. Association of senescent cells’ accumulation with tissue/organ pathology has been established for differentiated cells, while the effect of senescent stem cells on the capacity of tissues for regeneration has also been reported [17].

Given that chromatin structure plays a key role in cell regulation and organism lifespan [18,19], epigenetic alterations (i.e., DNA methylation, histone modification and chromatin remodeling) play a crucial role in the induction and maintenance of senescence and have been considered an additional hallmark of aging [1,12]. Especially DNA methylation profiles have been shown to be tissue- and cell-type specific, while overall, DNA methylation has been reported to decrease in replicative senescence, a pattern that has not been shown to be followed in prematurely senescent cells [20,21]. The association between DNA methylation and chronological age has been successfully used to create a series of epigenetic clocks estimating the acceleration of biological age in health and in pathological conditions, e.g., the primary DNAmAge predictor by Horvath et al. [22] or DNAmAgeHannum by Hannum et al. [21]. These clocks are based on methylation levels in specific CpG sites and not only reflect the advancement of biological processes, but also predict accompanying death risk [23].

With this study, we aim to provide a comprehensive characterization of DNA methylation patterns in replicative and premature senescence induced by two genotoxic stresses (i.e., exposure to ionizing irradiation and doxorubicin), exhaustively exploring the epigenetic modifications in three different human cell types, namely in somatic diploid skin fibroblasts and in bone marrow- and adipose-derived mesenchymal stem cells.

## 2. Materials and Methods

### 2.1. Experimental Design

The design of this study foreseen assessment of DNA methylation in five different conditions: in early-passage cells, in cells after a middle number of passages (MidPass), in replicative senescence (RS) and in stress-induced premature senescence (SIPS) after exposure to ionizing irradiation (IRR-SIPS) and doxorubicin (Dox-SIPS). In order to explore whether the epigenetic response is tissue-specific, all five treatments were applied to three different cell types: human diploid skin fibroblasts (DSF), human bone marrow-derived mesenchymal stem cells (hBM-MSC) and human adipose-derived mesenchymal stem cells (hAd-MSC). Samples were collected from six healthy individuals (two donors for each cell type), each donating only a single type of cell. Treatments of replicative senescence and SIPS were performed in duplicate for each donor.

### 2.2. Cells and Cell Culture Conditions

Primary human DSF, hBM-MSC and hAd-MSC deriving from consenting normal donors have been previously isolated in our laboratory and were retrieved to be used in the current study from our established cell bank. Primary human DSF have been isolated from dermal tissue explants, as described before [24]; primary hBM-MSC have been established after the immunomagnetic isolation of BM-CD105^+^ cells using Milteny microbeads according to the manufacturer’s instructions (Miltenyi Biotech, Bergisch Gladbach, Germany) [25]; primary hAd-MSC have been established after incubation of adipose tissue with 1.0 mg/mL of collagenase for 1 h. hBM-MSC and hAd-MSC have been characterized based on their ability for osteogenic, chondrogenic and adipogenic differentiation. Cells were routinely cultured in Dulbecco’s modified Eagle’s medium (DMEM) of 4.5 mg/mL and 1.0 mg/mL glucose for human DSF and hBM-MSC/hAd-MSC, respectively (PAN-Biotech, Aidenbach, Germany), supplemented with penicillin (100 U/mL), streptomycin (100 mg/mL) (obtained from Biosera, Nuaille, France) and 10% (*v*/*v*) fetal bovine serum (FBS) (from Gibco BRL, Invitrogen, Paisley, UK) in a humidified atmosphere of 5% CO_2_ and 37 °C. Cells were subcultured either when confluent (human DSF) or at 80% confluency (hBM-MSC and hAd-MSC) using a trypsin/citrate (0.25%/0.30% *w*/*v*) solution.

### 2.3. Induction of RS and SIPS

Replicative senescence of human DSF, hBM-MSC and hAd-MSC was achieved after serial subculturing of early-passage cells up to the exhaustion of their proliferative potential (approx. 65, 35 and 45 population doublings for human DSF, hBM-MSC and hAd-MSC, respectively). In order to induce SIPS, early-passage cells were either exposed to γ-irradiation in a ^60^Co gamma source (Gamma Chamber 4000A, Isotope Group, Bhadha Atomic Research Company, Trombay, Bombay, India) at a rate of 2.5 Gy/min, as previously described [26] or exposed to two non-cytotoxic doses of doxorubicin (0.1 μΜ/dose). Cells were then subcultured (usually twice) until their proliferative ability was exhausted. Establishment of RS and SIPS was confirmed by the inability of the cells to incorporate bromodeoxyuridine (BrdU) into their nuclei. Cells up to passage 5 were considered early-passage cells, while cells reaching half the total number of cell doublings (as that was estimated for each cell type) were considered middle-passage cells (MidPass).

### 2.4. Estimation of Bromodeoxyuridine (BrdU) Incorporation

The proliferative potential of the cells was estimated after labeling with 5-bromo-2’-deoxyuridine (BrdU), as previously described [27]. Briefly, cells were plated sparsely on sterile glass coverslips in DMEM containing 10% (*v/v*) FBS. BrdU (50 μM) was added to the cell culture medium for a period of 48 h. Cells were fixed with freshly prepared 4% (*v*/*v*) formaldehyde in phosphate-buffered saline (PBS) for 10 min, permeabilized with 0.2% (*v*/*v*) Triton X-100 in PBS for 10 min, treated with 2 N HCl for 30 min and incubated with an anti-BrdU-FITC antibody from BioLegend (SanDiego, CA, USA) overnight at 4 °C. Cells were then counter-stained with 2.0 μg/mL 4’,6-diamino-2-phenylindole (DAPI) dihydrochloride in PBS for 10 min. Labeled nuclei were observed under a Zeiss Axioplan 2 fluorescent microscope (Zeiss, Jena, Germany).

### 2.5. Cell Lysis and DNA Extraction

Cells were detached by trypsinization using a trypsin/citrate (0.25%/0.30% *w*/*v*) solution and were recovered by centrifugation at 500× *g* for 5 min. Cell pellets were washed once with PBS to remove any culture medium and FBS residuals and pellets were stored at −80 °C until genomic DNA extraction.

DNA extraction was performed using the NucleoSpin Tissue kit (Macherey-Nagel, Düren, Germany) according to the manufacturer’s instructions. In brief, cell pellets were lysed in the presence of Proteinase K at 70 °C for 10 min, and samples were applied to the NucleoSpin Tissue columns after the addition of 96–100% (*v*/*v*) ethanol. Silica membranes of the columns were washed twice and dried before elution of the bound DNA. DNA content was estimated using a Nanodrop ND-1000 spectrophotometer (Nanodrop Technologies, Wilmington, DE, USA).

### 2.6. DNA Methylation Assay

Genomic DNA was bisulfite-converted using the EZ DNA Methylation Kit (Zymo Research) and analyzed using the Infinium Human MethylationEPIC BeadChip (Illumina) according to the respective manufacturer’s instructions.

### 2.7. Data Exploration

Principal component analysis (PCA) was used as an exploration tool to discover the patterns present in DNA methylation data and to aid data interpretation [28] since it captures the major sources of variation in the data and helps combine the traits and identify the main attributes with high likelihood of comprising the differences in methylation patterns necessary to distinguish groups of samples. The normalized dataset’s dimensions were reduced with PCA using *prcomp()* function provided by *stats* R package, and the obtained components were further used to perform heat map and cluster analysis to visualize the grouping patterns present in the data.

Heat maps were created with the support of the *heatmap()* function of *stats* R package to identify the distribution of associations between individual samples (represented on the vertical axis of the graph) and a number of components retrieved from PCA (on the horizontal axis). Very low and very high values of principal components (PCs) were represented by extreme colors ranging from light yellow to dark red, respectively. Heat maps were complemented with dendrograms on the chart sides, visualizing the arrangement of sample clusters and helping to identify the objects with the highest and lowest similarity. The depth of sample clustering—expressing, at the same time, the level of considered similarity/dissimilarity—was marked by a purple vertical line. Sample clustering was highlighted by colored boxes grouping the samples with a similar methylation profile.

### 2.8. Data Analysis

Output idat files from the Illumina platform were parsed, and raw signal intensities in the green and red channels were extracted using *minfi* package within R Bioconductor software. After calculation of CpG sites’ detection *p*-value per CpG site, quality control was performed and poor-quality samples—specifically those with mean detection *p*-value above 0.05—were removed. In order to remove undesired variations in the data, normal exponential convolution using the out-of-band Infinium I probes (*Noob*) normalization method was applied. Further filtering excluded probes that (i) failed in at least one of the samples (detection *p*-value > 0.01), (ii) were located on X- or Y-chromosomes, (iii) mapped to SNPs and iv) were multiple-aligning, cross-reactive or masked from mapping.

### 2.9. Differential Methylation Analysis

#### 2.9.1. Global Methylation Levels

Changes in global methylation levels observed in four different conditions—MidPass, RS, IRR-SIPS, Dox-SIPS—were assessed comparing β-values in “after treatment” and reference early-passage cultures. Mean methylation difference was determined as an average value of ∆β = β_Treatment_ − β_Reference_ calculated for all the probes. A positive value of mean methylation difference (Avrg(∆β) > 0) indicated hypermethylated state in treated compared to reference cells, while a negative value indicated hypomethylated state. Paired *t*-test and subsequent multiple testing correction with Benjamini and Hochberg’s (BH) method were used to evaluate if detected alterations were statistically significant. α = 0.05 was defined as the level of statistical significance.

#### 2.9.2. Differentially Methylated Positions

*DBSCAN* algorithm was used to exclude methylation probes that had a bi- or tri-modal trend among control subjects of the same sex [29]. Probe-wise approach was applied to identify local alterations in the DNA methylation patterns; thus, multiple linear models were created using ordinary least squares fitting (*limma* R package) for each CpG using methylation *M*-values. In the models, Donor was included as a random effect. Results of the fitting were used in hypothesis testing performed with the robust empirical Bayes procedure. Test statistics were adjusted for possible bias and inflation using *bacon* [30]. *p*-values were adjusted for multiple testing with the BH approach. Differentially methylated positions (DMPs) were defined as CpG sites that reached statistical significance level with BH-corrected *p*-value < 0.05 and for which the absolute difference between mean β-values (∆β) of two compared phenotypic groups was above 20%.

Cluster analysis was employed to separate samples with dissimilar DNA methylation profiles and recognize highly similar groups based on the emerged DMPs. For this purpose, PCA was performed on the reduced dataset comprising β-values exclusively of significant CpGs, and the *fviz_pca_ind()* function provided within *factoextra* R package was used for visualization. In the graphical representation, the first and second PCs explaining the greatest percentage of the total variability of a studied phenotype were plotted, respectively, on the vertical and horizontal axis. The colors of plotted individuals corresponded to their quality of representation on the cluster map (*cos2*).

### 2.10. Senescence-Related External Resources

In order to support our findings, to comment on results obtained with differential methylation analysis and to complete the interpretation, external online resources dedicated to senescence were consulted. SeneQuest (available at http://Senequest.net; accessed on 28 April 2022) is a comprehensive resource tool gathering and summarizing the information on gene-to-senescence associations emerging from up-to-date publications [31], while Human Aging Genomic Resources, HAGR (available at https://www.genomics.senescence.info/; accessed on 27 November 2020) is a rich repository of specialized databases and tools supporting research on aging [32,33].

### 2.11. Pathway Enrichment Analysis

Emerged DMPs were annotated with genes and sets of unique genes involved in DNA methylation alterations observed in treatments with respect to early-passage cultures. Pathway enrichment analysis was performed employing *Enrichr* web-based tool [34,35] that allowed annotation of revealed gene lists with frequently occurring pathways using the KEGG database [36,37]. We focused on the pathways for which the *p*-value from Fisher’s exact test reached a statistical significance level < 0.05.

Since pathway enrichment analysis revealed an intriguingly high number of KEGG terms related to nervous system (NS), we extended this analysis to verify if the number of significant NS-related pathways is indeed statistically increased compared to other returned entities. Thus, *neuro*-pathway enrichment analysis was performed. For this purpose, a reference list of *neuro*-pathways was created, including pathways widely related to nerves, nervous system and neurological mechanisms. The list comprised all the terms indicated in the following KEGG map classes: “signal transduction”, “signaling molecules and interaction”, “nervous system”, “sensory system”, “development and regeneration” and “neurodegenerative diseases”. The list was extended with the selective addition of “endocrine system” maps. The final set of terms used for annotation is provided in Supplementary Information Table S1. Thus, lists generated in pathway analysis were reviewed and annotated with their eventual involvement in the broadly understood neurological context. Fisher’s exact test (right-sided) was employed in order to estimate statistical significance of the enrichment in *neuro*-pathways.

### 2.12. DNA Methylation Clocks Analysis

Whole-genome DNA methylation data were used to evaluate the epigenetic age of samples with Horvath’s new DNA Methylation Age Calculator available online (https://dnamage.genetics.ucla.edu/; accessed on 30 July 2021 ). The DNA methylation-based predictor of biological aging DNAmAge, a universal model for many tissues and organs developed on 353 CpG sites, was calculated for human DSF, hBM-MSC and hAd-MSC samples [22]. Two-way ANOVA and post-hoc Tukey’s HSD test were applied to detect the presence of differences in epigenetic ages among distinct cell states using 0.05 as the significance *p*-value threshold.

## 3. Results

Human DSF, hBM-MSC and hAd-MSC were collected from six healthy subjects according to the experimental design. Each individual was a donor only of a single cell type. Except for DSF2, all of the recruited subjects were females. Cells that exhibited less than 3% of BrdU incorporation were considered senescent (data not shown).

**Figure 1:**
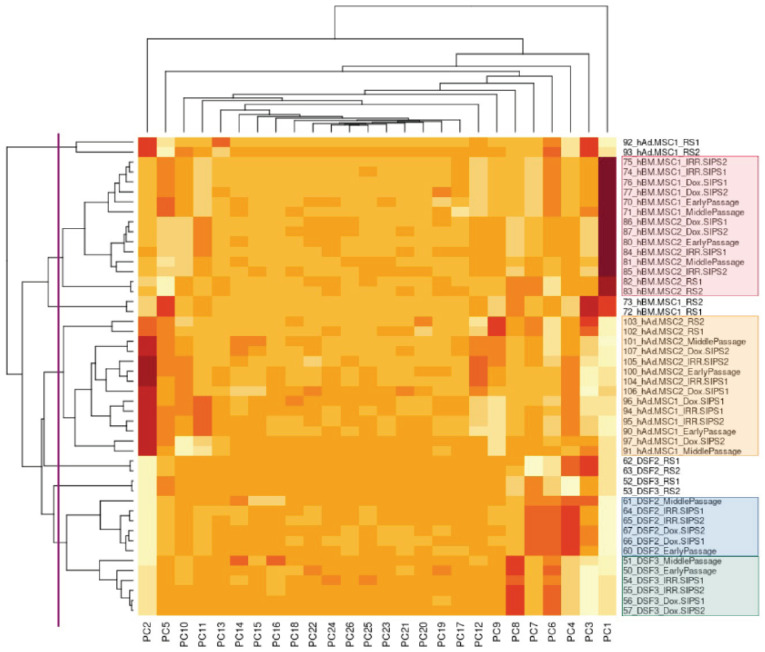
Heat map of principal component analysis (PCA) of DNA methylation data for the entire studied cohort. Vertical axis corresponds to analyzed samples: on the right, sample names are listed, while on the left, the dendrogram of sample clustering is plotted. Horizontal axis corresponds to principal components (PCs): on the bottom, the 26 components used are specified, while on the top, the dendrogram of PC clustering is presented. Purple vertical line marks the depth of sample clustering considered, and colored blocks highlight the obtained sample groups. On right, sample names are listed (numbers from 51 to 106) with indication of cell origin (DSF, hBM-MSC, hAd-MSC) and sample condition (EarlyPassage, early-passage cells; MiddlePassage, cells after a middle number of passages; RS, cells in replicative senescence; IRR.SIPS, cells in stress-induced premature senescence after exposure to ionizing irradiation; Dox.SIPS, cells in stress-induced premature senescence after exposure to doxorubicin).

To explore DNA methylation data and discover similarity–dissimilarity patterns among samples and treatments, normalized β-values for the entire cohort were subjected to PCA and the first 26 components explaining the highest percentage of variability in the dataset were used for visualization. Samples tended to cluster within the cell type, as highlighted by the colored blocks on the right side of Figure 1. In all human DSF, hBM-MSC and hAd-MSC, RS seemed to cause major changes in the methylation pattern, leading always to distinct epigenetic profiles.

### 3.1. Differential Methylation Analysis

In total, DNA methylation was assessed in 48 samples, specifically in 16 originating from human DSF, 16 from hBM-MSC and 16 from hAd-MSC. For each cell type, five conditions were available: Early-passage, Middle-passage, RS, IRR-SIPS and Dox-SIPS. Early-passage cell cultures were considered as a reference state to identify epigenetic alterations, and all four other conditions are referred here as the treatment states.

#### Global Methylation Levels

We began the exploration of the collected data with the question of whether there are cell type-specific or global epigenome alterations characterizing any of the treatments. The results of our analysis are summarized in Table 1. In all cell types, samples after a middle number of passages presented global hypomethylation when compared to early-passage cells, with the mean difference in methylation levels being equal to −0.0044, −0.0004 and −0.0103 for fibroblasts, hBM-MSC and hAd-MSC, respectively. Compared to early-passage cells, replicative senescent cells were found to be hypermethylated in fibroblasts (with an average ∆β equal to 0.0057) and hypomethylated in both types of mesenchymal stem cells (with the average difference reaching −0.0053 and −0.0202 for hBM-MSC and hAd-MSC, respectively). Prematurely senescent cells (IRR-SIPS and Dox-SIPS), on the other hand, were found hypermethylated in comparison to early-passage cells in all cell types investigated (mean ∆β reaching 0.0066 and 0.0056 in IRR-SIPS and Dox-SIPS, respectively, for fibroblasts; 0.0071 and 0.0035 in IRR-SIPS and Dox-SIPS, respectively, for hBM-MSC; and 0.0034 and 0.0038 in IRR-SIPS and Dox-SIPS, respectively, for hAd-MSC). All reported differences in global methylation remained significant after BH-correction for multiple testing (adjusted *p*-value < 0.05).

### 3.2. Cell Type-Specific & Treatment-Specific Epigenetic Signatures

The second question in our study–description of simultaneous cell type- and cell condition-specific epigenetic signatures–was addressed, identifying exclusive sets of DMPs summarized in Table 2. The full lists of differentially methylated sites are provided in Supplementary Information Tables S2–S13. Emerged sites were mapped to genes and allowed the creation of lists of unique genes for which at least one significant CpG site (DMP) was found.

**Figure 2:**
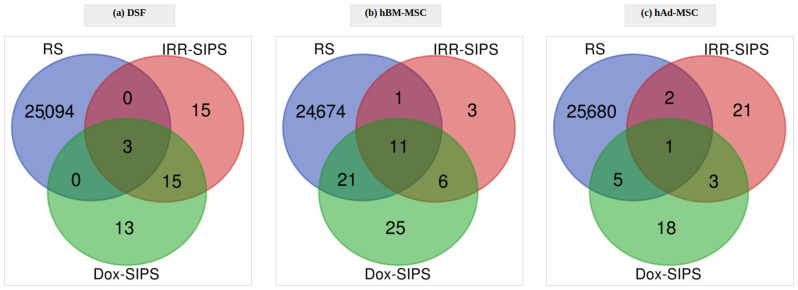
Differentially methylated position overlaps among the three types of senescence (replicative senescence RS, stress-induced premature senescence after exposure to ionizing irradiation IRR-SIPS and doxorubicin Dox-SIPS) in (**a**) DSF, (**b**) hBM-MSC and (**c**) hAd-MSC.

In human DSF, 636 differentially methylated CpGs were detected when comparing samples from early-passage and MidPass conditions, which corresponded to 366 unique genes. In RS, the number of DMPs was considerably higher than in other conditions, reaching 25,097 significant sites distributed across 9486 genes. Stress-induced senescence resulted in similar alterations of epigenetic profiles for both irradiation and doxorubicin treatment, reaching 33 and 31 significant CpGs, respectively (for both cases, 25 genes). This similarity between stressors was manifested, however, not only in the number of DMPs and genes, but also in the overlap between particular sets, reaching almost 60%: 18 and 14 common CpGs (Figure 2a) and genes (Supplementary Information Figure S1a), respectively.

Analysis of data from hBM-MSC cultures revealed 64 CpG sites and 45 unique genes that had methylation levels significantly different between MidPass and early-passage cells. RS-related epigenetic modifications included 24,707 DMPs mapping to 7884 genes. Furthermore, 21 CpGs (16 genes) and 63 CpGs (44 genes) were identified as differentially methylated after irradiation and treatment with doxorubicin, respectively, with 17 DMPs (Figure 2b) and 12 genes (Supplementary Information Figure S1b) being common between IRR- and Dox-SIPS.

In samples originating from hAd-MSC, the signal that was found significant in MidPass compared to early-passage cells reached 1426 sites spread over 829 genes. Epigenetic modifications observed in RS included 25,688 DMPs and 7422 genes. Additionally, 27 differentially methylated CpGs and 19 unique genes in IRR-SIPS treatment were identified. Similarly, in Dox-SIPS, 27 DMPs corresponding to 23 genes were detected; however, the overlap between signatures of both stress-induced senescent states was limited to four significant CpG sites (Figure 2c) and three genes (Supplementary Information Figure S1c).

**Figure 3:**
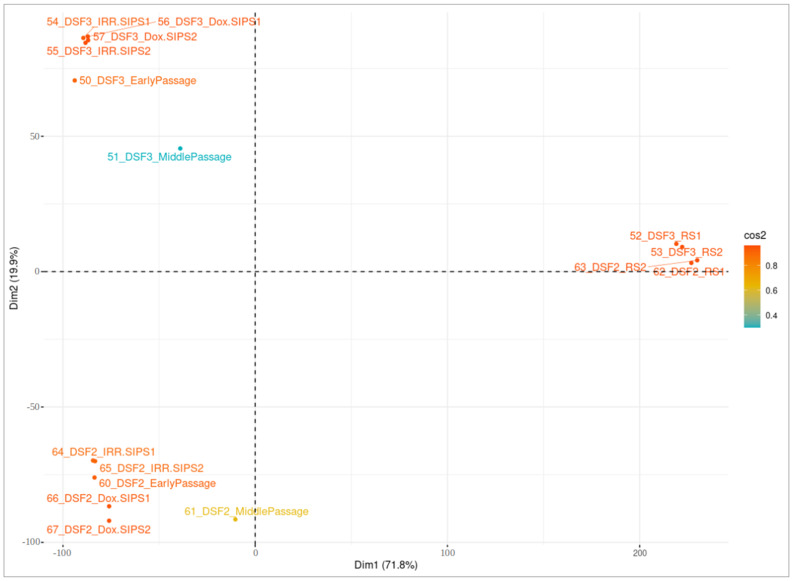
Visualization of cluster analysis in human DSF samples. Principal component analysis (PCA) was performed on normalized β-values of unique differentially methylated positions in cells after a middle number of passages (MidPass), in replicative senescence (RS), in stress-induced premature senescence after exposure to ionizing irradiation (IRR-SIPS) and doxorubicin (Dox-SIPS). X-axis “Dim1” corresponds to the first principal component of PCA, and Y-axis “Dim2” to the second principal component. The percentage of total variability explained by the component is indicated in brackets. Colors reflect the quality of representation on the cluster map (cos2). Labels correspond to samples (for detailed explanation of sample indicators, consult Figure 1).

**Figure 4:**
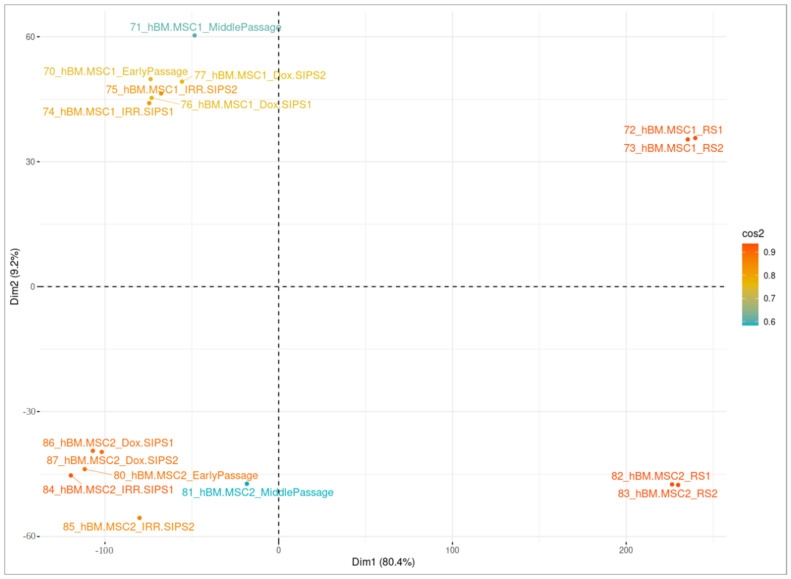
Visualization of cluster analysis in hBM-MSC samples. Principal component analysis (PCA) was performed on normalized β-values of unique differentially methylated positions in cells after a middle number of passages (MidPass), in replicative senescence (RS), in stress-induced premature senescence after exposure to ionizing irradiation (IRR-SIPS) and doxorubicin (Dox-SIPS). X-axis “Dim1” corresponds to the first principal component of PCA, and Y-axis “Dim2” to the second principal component. The percentage of total variability explained by the component is indicated in brackets. Colors reflect the quality of representation on the cluster map (cos2). Labels correspond to samples (for detailed explanation of sample identifiers, consult Figure 1).

**Figure 5:**
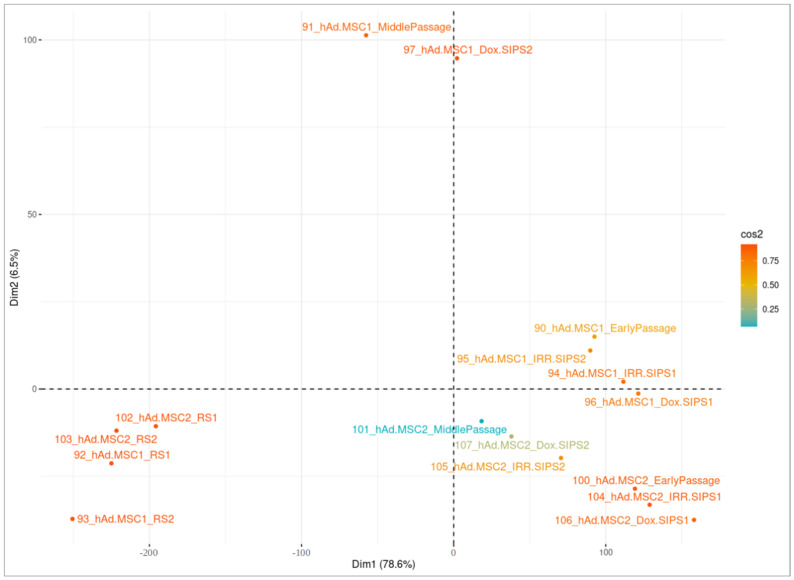
Visualization of cluster analysis in hAd-MSC samples. Principal component analysis (PCA) was performed on normalized β-values of unique differentially methylated positions in cells after a middle number of passages (MidPass), in replicative senescence (RS), in stress-induced premature senescence after exposure to ionizing irradiation (IRR-SIPS) and doxorubicin (Dox-SIPS). X-axis “Dim1” corresponds to the first principal component of PCA, and Y-axis “Dim2” to the second principal component. The percentage of total variability explained by the component is indicated in brackets. Colors reflect the quality of representation on the cluster map (cos2). Labels correspond to samples (for detailed explanation of sample identifiers, consult Figure 1).

We performed cluster analysis separately for each cell type using the sets of unique significant CpGs in each studied condition, and the results are presented in Figure 3, Figure 4 and Figure 5. Overall, there were 25,604, 24,785 and 26,335 unique DMPs in human DSF, hBM-MSC and hAd-MSC, respectively, as summarized in Supplementary Information Table S17. For all three cell types, samples of a single donor tended to cluster together and separate from distinct donors, indicating that the interpersonal variability was higher than the treatment-related intrapersonal variability. Only RS samples created an isolated cluster reflecting notable alterations in the DNA methylation patterns accompanying RS. Additionally, RS cells seemed to lose their donor-specific epigenetic identity since they tended to be approximal within the space of the clustering graph, surpassing interpersonal dissimilarities. It is worth noting that MidPass cells seemed to represent an interstitial state between early-passage and RS cells since, in the plot space, they presented a drift from early-passage/IRR-SIPS/Dox-SIPS clusters in the direction of RS groups.

#### 3.2.1. Cell Type-Specific Senescence Epigenetic Signatures

Next, we inquired into epigenetic alterations common for any type of senescence found simultaneously in RS, IRR-SIPS and Dox-SIPS conditions for each cell type. According to the obtained results, the overlaps were limited, as summarized in Figure 2. In human DSF, three DMPs were shared among all three treatments, of which two were located in genic regions within *POU2F3* and *TMC1* genes. Both genes have been found to be overexpressed in human cell lines during senescence, according to SeneQuest. Eleven common differentially methylated sites were detected in hBM-MSC mapping to *MCCC1*, *LOC101928008*, *SBF2*, *FGF8*, *MIER1*, *DIAPH3*, *RAD51B*, *ZNF438* and *TANC1* genes. According to SeneQuest, *DIAPH3* and *RAD51B* have been previously reported to be downregulated in senescent human cells, while for *ZNF438* and *TANC1,* up- and downregulation have been reported in different cell lines. *TANC1* was the only gene present also in the HAGR database, found as overexpressed in cellular senescence. In hAd-MSC, a single CpG reached statistical significance for all three senescence treatments and was located in the *MIER1* gene that has not been previously linked to senescence, according to the SeneQuest or HAGR database. Overlaps between IRR-SIPS and Dox-SIPS tended to be higher than those with RS, indicating that both types of stress-induced senescence share common epigenetic mechanisms.

#### 3.2.2. Treatment-Specific Epigenetic Signatures

**Figure 6:**
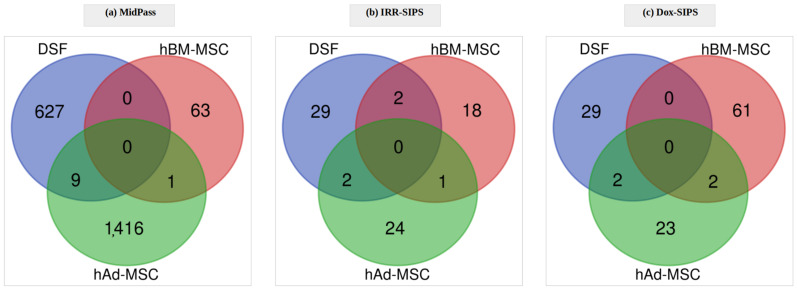
Differentially methylated positions shared among the three cell types after (**a**) a middle number of passages (MidPass), (**b**) irradiation-induced senescence (IRR-SIPS) and (**c**) doxorubicin-induced senescence (Dox-SIPS).

**Figure 7:**
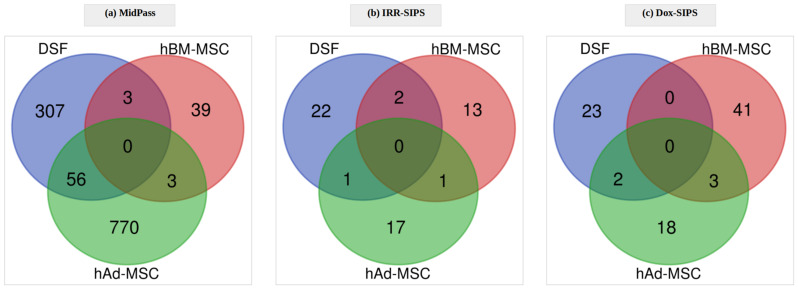
Genes shared among the three cell types after (**a**) a middle number of passages (MidPass), (**b**) irradiation-induced senescence (IRR-SIPS) and (**c**) doxorubicin-induced senescence (Dox-SIPS).

Further, we searched for modifications in DNA methylation patterns that would be treatment-specific but transversal across the three cell types. Neither significant CpGs (Figure 6) nor genes (Figure 7) were shared among human DSF, hBM-MSC or hAd-MSC cells in MidPass, IRR-SIPS and Dox-SIPS conditions. There was a single common gene between IRR- and Dox-SIPS and between hBM- and hAd-MSC: *MIER1*, which has not been previously associated with senescence based on the entries in SeneQuest and HAGR databases.

**Figure 8:**
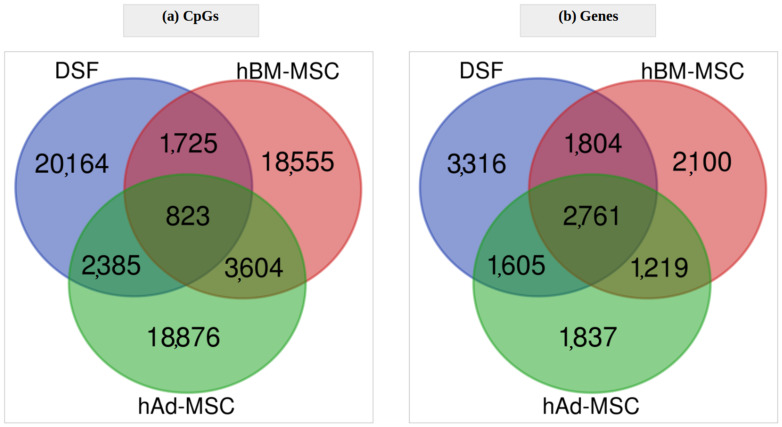
Overlap of differentially methylated (**a**) CpG sites and (**b**) genes in replicative senescence of all three cell types: DSF, hBM-MSC and hAd-MSC.

On the other hand, in RS, as illustrated in Figure 8a, 823 DMPs (3%) were common to all assessed cell types. Considering, instead, lists of the genes, the overlap included 2761 items counting for 30–37% of the cell-specific gene sets (Figure 8b). Among the overlapping entities were found genes such as *TP63*–Tumor Protein P63, *XAF1*–XIAP-Associated Factor 1, SLC13A3–Solute Carrier Family 13 Member 3 or *EZH2*–Enhancer Of Zeste 2 Polycomb Repressive Complex 2 Subunit. The fact that the number of common genes exceeded so remarkably the number of common differentially methylated CpGs confirms that epigenetic mechanisms implicated in replicative senescence, independent of the cell type, do not rely on single CpG sites but rather on extended alterations spread over genes becoming epigenetic players. In other words, alterations in the DNA methylation status of one or several adjacent CpG sites plausibly produce equivalent epigenetic outcomes. These sites create an “epigenetic effector unit” and tend to be located within a region of a single gene.

### 3.3. Pathway Enrichment Analysis

#### 3.3.1. Cell Type-Specific and Treatment-Specific Analysis

Genes that emerged from the annotation of DMPs identified in differential methylation analysis were further used in pathway enrichment analysis. The number of KEGG pathways found for the three cell types in each tested condition is summarized in Table 3.

In human DSF, after a middle number of passages, irradiation- and doxorubicin-induced senescence 17, 8 and 1 pathways were significant with nominal *p*-values (Table 3). None of them reached statistical significance level when considering the adjusted *p*-value (Table 3). In replicative senescent human DSF 10 of 45 significant pathways remained significant after correction for multiple testing, and they are described in detail in Table 4.

In hBM-MSC, after a middle number of passages, there was a single KEGG term with a nominal *p*-value < 0.05, but it did not remain significant after correction for multiple testing (Table 3). In senescent cells 33, 14 and 20, significant pathways were identified for RS, IRR-SIPS and Dox-SIPS, respectively, that were reduced to 5, 2 and 4 terms reaching significance level also with adjusted *p*-value (Table 3). Details on pathways remaining significant after *p*-value correction are provided in Table 5.

Data collected from hAd-MSC revealed 57 and 43 pathways with significant nominal *p*-values in MidPass and RS, respectively (Table 3). After correction for multiple testing, seven and eight terms remained significant (Table 3), and the details are presented in Table 6. Eleven pathways were found to be enriched in irradiation-induced senescence, and seven after the treatment with doxorubicin. None of the identified items reached the significance level when the adjusted *p*-value was considered (Table 3).

Comprehensive results of pathway analysis with complete lists of pathways that reached significance with nominal *p*-values are provided in Supplementary Information Tables S14–S16.

#### 3.3.2. Treatment-Specific Analysis

Due to the number of genes identified as common for the three cell types and specifically for the particular tested conditions, only the set that emerged from the analysis of replicative senescence allowed for further pathway enrichment analysis. As a result, a list of 83 pathways that reached statistical significance with a nominal *p*-value was obtained. Fifty-one of the KEGG terms remained significant after correction for multiple testing, and they are presented in Table 7.

As presented in Table 7, the performed pathway enrichment analysis returned a number of KEGG terms linked to the human nervous system (NS). In order to verify if the observed enrichment in NS-related entities was statistically significant, our study was complemented with *neuro*-pathway enrichment analysis performed as described in the Materials and Methods section. Fisher’s exact test was applied to examine whether the KEGG map obtained from a selection of common RS-related genes contained an increased number of significant pathways related to a broadly considered neuro-context, comparing the non-significant terms. Considering as significant those KEGG terms that reached adjusted *p*-value below 0.05, we obtained a contingency table with 25 significant and 41 non-significant NS-related pathways in comparison to 25 significant and 212 non-significant non-NS-related pathways. As a result, Fisher’s exact test returned a *p*-value of 9.70 × 10^−7^, indicating significant enrichment in *neuro*-pathways. If the significance of KEGG terms was determined based on their nominal *p*-values instead, the contingency table contained 36 significant and 30 non-significant NS-linked pathways in contrast to 47 and 90 significant and non-significant non-NS-related pathways, respectively. Thus, Fisher’s exact test returned a *p*-value of 8.73 × 10^−8^, confirming again the statistical significance of neuro-pathway enrichment.

### 3.4. DNA Methylation Clocks Analysis

**Figure 9:**
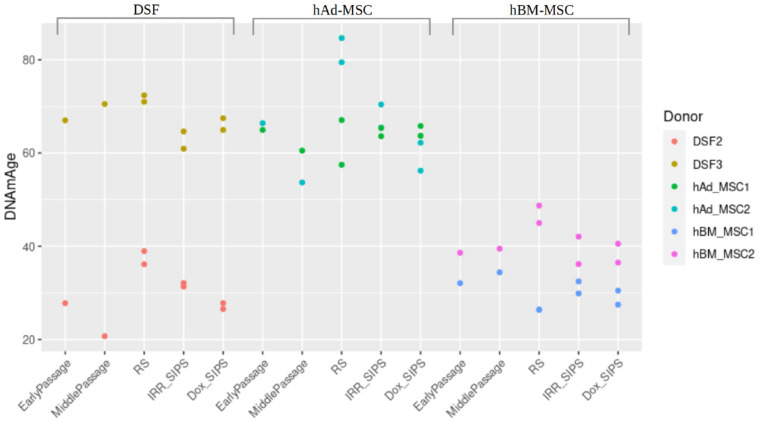
DNA methylation-based prediction of biological age using Horvath’s DNAmAge model in human DSF, hAd-MSC and hBM-MSC from different cell cultures: early-passage cells (EarlyPassage), cells after a middle number of passages (MiddlePassage), cells in replicative senescence (RS), cells in stress-induced premature senescence after exposure to ionizing irradiation (IRR_SIPS) and cells in stress-induced premature senescence after exposure to doxorubicin (Dox_SIPS).

The acceleration of biological age was evaluated in human DSF, hBM-MSC and hAd-MSC using Horvath’s DNAmAge model. Even though no statistically significant differences in the epigenetic age of early-passage, MidPass, RS, IRR-SIPS and Dox-SIPS cells were found, a trend was observed in all three cell types; that is, replicative senescent cultures tended to present higher values of predicted biological age than cultures of the four other conditions (Figure 9).

## 4. Discussion

Aging is characterized by the inevitable progressive decline in tissue and organ function and the increased risk for morbidities and mortality. Both falling within the hallmarks of aging, cellular senescence and epigenetic alterations are interconnected, given that epigenetic dysregulation has been considered a key driver for cellular senescence and stem cell aging [38,39,40]. DNA methylation, in particular, has been reported to change during cellular senescence in a context-dependent manner [38]. For example, altered DNA methylation patterns have been observed in replicative senescent cells, but not in prematurely irradiation-, oncogene- and non-permissive temperature-induced senescent cells [20,41]. Given that the epigenetic mechanisms underlying replicative and stress-induced premature senescence have not yet been fully elucidated, the aim of the current study was to investigate (i) global DNA methylation, (ii) epigenetic signatures and (iii) biological age acceleration in three different types of cellular senescence in human DSF, as well as in hBM-MSC and hAd-MSC. For each cell type, samples from two individual donors were analyzed. Senescence in cells was induced as a result of replicative exhaustion due to long-term serial subculturing or as a response to genotoxic stress, i.e., exposure to ionizing irradiation and doxorubicin.

The obtained results confirmed the presence of alterations in global DNA methylation patterns. Global hypo- and hyper-methylation were consistently found in all three cell types in cultures after a middle number of passages and in irradiation-/doxorubicin-induced prematurely senescent cells, respectively. In replicative senescence instead, human DSF were hypermethylated compared to the respective early-passage cultures, while in both mesenchymal stem cells, global DNA hypomethylation was observed. In our work, we identified three epigenetic signatures: (a) cell type- and treatment-specific signature; (b) cell type-specific senescence-related signature; and (c) cell type-transversal replicative senescence-related signature. Cluster analysis performed on methylation data of cell-specific DMPs for all treatments demonstrated high interpersonal variability of epigenetic profiles and increased similarity in the methylation patterns of early-passage, IRR-SIPS and Dox-SIPS cells. Replicative senescence is accompanied by a profound epigenetic remodeling to the point that eventually surpasses interpersonal dissimilarities. Middle-passage cells present a methylation profile that is between replicative senescent and early-passage cells, suggesting that epigenetic remodeling is a progressive process. However, our experimental design does not allow us to understand whether epigenetic modeling is a consequence or a cause of the replicative senescence process. Nevertheless, it is reasonable to assume that this profound methylation reshaping led to consistent changes in the molecular physiology of the cells, eventually contributing to the replicative senescence phenotype. From the observation of the genes emerging from the cell type-transversal replicative senescence-related signature, we noted in many comparisons an enrichment in pathways linked to the nervous system functions. We performed Fisher’s exact test to confute this observation, and the analysis confirmed that neuro-system function is significantly enriched with respect to other pathways. These pathways indeed contained an increased number of significant KEGG terms involved in “signal transduction”, “signaling molecules and interaction”, “nervous system”, “sensory system”, “development and regeneration”, “neurodegenerative diseases” and “endocrine system”. Taking together these results led us to speculate that, in consideration of the high epigenetic homogenizing effect of cellular senescence on the different cell types, such common methylation remodeling could be particularly detrimental in the cells belonging to the nervous system, in agreement with the theory that the cellular senescence process plays a role in neurodegenerative diseases [42]. Regarding biological age, replicative senescent cells of all types tended to present higher values of predicted biological age, without reaching statistical significance that would evidence the acceleration of DNAmAge.

The decrease in global methylation levels during in vitro culturing of animal and human cells has been reported [43,44,45,46,47], and a set of focal site-specific alterations has been described [43,48,49]. In the study of Bork et al., DNA methylation was investigated in replicative senescent hBM-MSC [43]. In contrast to our study, Bork et al. detected no changes in the global methylation patterns of early- and late-passage cultures. A number of sites associated with RS were identified, particularly 29 hypermethylated CpGs (corresponding to 28 unique genes) and 55 hypomethylated CpGs (mapping to 51 unique genes). We also found 3 of the above-mentioned 29 and 5 of the above-mentioned 55 sites to be RS-related DMPs in hBM-MSC. When considering the lists of unique genes, the specific overlaps between the results of both studies reached 13 of 28 (i.e., *LCAT*, *CPA1*, *DLX5*, *MAMDC2*, *FES*, *ACTA2*, *FGFR1*, *SPARCL1*, *MYF5*, *TRIM65*, *RUNX3*, *TSC1*, *ISLR*) and 21 of 51 items (i.e., *PRSS1*, *RAB24*, *DSG4*, *CEACAM3*, *SCN7A*, *KRTAP26-1*, *C18orf20*, *SPRR2A*, *CTSG*, *VN1R2*, *PBOV1*, *DPP6*, *LY9*, *CCR3*, *REG3A*, *LACRT*, *SPRR3*, *GLIS1*, *RUNX3*, *MYH1*, *KRTAP11-1*), respectively. Some of these genes have already been linked to serious neurological conditions, such as *SCN7A,* which has been associated with amyotrophic lateral sclerosis [50], *GLIS1* with Parkinson’s Disease [51] and *SPARCL1* with neuroinflammation in Alzheimer’s Disease [52]. Pathways related to the discovered genes in the work of Bork et al. were enriched, particularly in processes related to development. In the cited study, authors identified additional age-related changes by comparing DNA methylation in early-passage cells derived from young (21–50 years old) and elderly (53–85 years old) donors, and they verified the presence of overlaps between both senescence- and age-associated signatures.

Koch et al. investigated culture expansion-related DNA methylation alterations in dermal fibroblasts, mesenchymal stem cells from bone marrow and adipose tissue [53]. According to the authors, unsupervised PCA revealed two tendencies: clustering of the cells deriving from the same tissue and distinction of the respective RS cultures in accordance with our findings. Changes in the methylation pattern were shown to be correlated with the number of passages, having a reproducible character and spreading constantly with the successive expansion steps. CpG sites associated with long-term culturing were predominantly hypermethylated. The authors focused particularly on six differentially methylated sites located in *GRM7* and *CASR* (both found hypermethylated), *PRAMEF2*, *SELP*, *CASP14* and *KRTAP13-3* genes (all found hypomethylated). These CpGs served to create an Epigenetic-Senescence-Signature model that was successfully validated in several cell types. Four of the six afore-mentioned genes, namely *GRM7*, *CASR*, *SELP* and *CASP14*, were found to be hypermethylated in replicative senescent human DSF, hBM-MSC and hAd-MSC in our study, compared to their respective early-passage cultures.

Another study further investigated senescence-related methylation changes by comparing methylomes of replicative senescent human dermal fibroblasts and human bone marrow-derived mesenchymal stem cells [49]. The authors confirmed the tendency in the clustering between the methylation profiles of cultures deriving from the same cell tissue. Similar to our observations, authors noticed that RS-related signatures for different cell types were considerably overlapping, indicating that epigenetic regulation of involved mechanisms must be at some part common and cell type-independent.

Koch and colleagues further explored senescence-related methylation changes in mesenchymal stem cells from human bone marrow [41]. Authors of this study found 1702 hypermethylated (corresponding to 1219 unique genes) and 2116 hypomethylated CpG sites (mapping to 1260 unique genes) in late-passage cultures, when compared to early-passage cells. Of these, 64 and 397 probes, respectively, were present in the list of RS-associated DMPs in hBM-MSC in our study. In terms of unique genes, the overlap with our work was 739 and 889 items, respectively. Furthermore, although exposure to irradiation resulted in the senescence of the cells, Koch et al. did not manage to identify any differentially methylated CpGs. Thus, DNA methylation could not be considered a causative mechanism in IRR-SIPS. This observation is in agreement with our results, since irradiation of hBM-MSC resulted in only 14 CpGs that reached the significance level with a nominal *p*-value, which was further limited to two sites that remained significant after correction for multiple testing.

The work of Bielak-Zmijewska et al. provided insight into the methylation alterations after Dox-SIPS treatment [54]. This study examined the epigenetics of senescence in vascular smooth muscle cells derived from the human aorta. Although the authors observed global DNA hypomethylation after replicative senescence, no change after doxorubicin treatment was detected. This observation is not in line with what could be expected since doxorubicin was shown to inhibit the enzymatic activity of *DNMT1*, leading, in turn to the downregulation of methylating events [55]. Additionally, the study confirmed an association between the number of culture passages and the shortening of telomeres, while Dox-SIPS seemed to have no effect on telomere length. Although—as reported above—there is evidence of some similarity in the epigenetic mechanisms among different cell types, a comparison of the results from the study of Bielak-Zmijewska et al. with ours should be conducted with caution, due to the different origin of the cells.

In the existing literature, there is a limited number of works on DNA methylation-based age in cellular senescence. Lowe et al. have explored the acceleration of the biological clock estimated with Horvath’s algorithm in senescent human coronary artery-derived endothelial cells [56]. Authors of the particular work confirmed acceleration of the epigenetic aging in RS and oncogene-induced senescence; however, no evidence of a similar tendency in irradiated cells was provided. Again, any discrepancy with our results could be attributed to cell-type-specific differences.

Interestingly, some of the genes identified in our methylation study are included in the CellAge signature of the HAGR repository, for instance, *TP63*, shown to induce senescence in mouse- and human-derived fibroblasts [57]; *XAF1*, found over-expressed in IRR-SIPS and Dox-SIPS pulmonary microvascular endothelial cells [58]; *SLC13A3*, accelerating RS in human diploid cells and renal tubular cells [59]; or *EZH2,* the deficiency of which has been shown to lead human gastric cancer cells to senescence [60]. Others could be retrieved from the SeneQuest senescence-focused search engine, such as *POU2F3* [61] and *TMC1* [62], which have been reported to display senescence-associated upregulated expression, or such as *DIAPH3* [61] and *RAD51B* [63] that have been reported to be downregulated in human cell lines. Previously reported data on the regulation of the discovered genes at the expression level could indicate that the epigenetic changes revealed in this study may be plausibly translated to transcriptomic events and may have functional consequences in the cells.

Overall, our work demonstrates changes in the DNA methylation levels of senescent human fibroblasts and stem cells, i.e., of human DSF, hBM-MSC and hAd-MSC. Epigenetic alterations varied among the cell types studied, while in all cases, they were more pronounced in replicative senescent cells than in cells rendered senescent after their exposure to ionizing irradiation or doxorubicin. This finding strongly supports the heterogeneity of senescent cells depending on the tissue of origin and the stimulus provoking senescence, implying diverse biological roles in tissue homeostasis and the development of age-associated diseases. Further characterization of these epigenetic signatures that could be linked to different degrees of sensitivity towards senotherapeutic compounds may prove extremely useful for designing novel therapeutic strategies.

## Figures and Tables

**Table 1:** Global methylation levels found in particular cell types under different conditions. Respective early-passage cultures were used as the reference.

Cell Type	Condition	Average (∆β)	*p*-Value	BH-Adjusted *p*-Value	Methylation State
**DSF**	MidPass	−0.0044	<2.2 × 10^−16^	0.000	Hypomethylation
	RS	0.0057	<2.2 × 10^−16^	0.000	Hypermethylation
	IRR-SIPS	0.0066	<2.2 × 10^−16^	0.000	Hypermethylation
	Dox-SIPS	0.0056	<2.2 × 10^−16^	0.000	Hypermethylation
**hBM-MSC**	MidPass	−0.0004	<2.2 × 10^−16^	0.000	Hypomethylation
	RS	−0.0053	<2.2 × 10^−16^	0.000	Hypomethylation
	IRR-SIPS	0.0071	<2.2 × 10^−16^	0.000	Hypermethylation
	Dox-SIPS	0.0035	<2.2 × 10^−16^	0.000	Hypermethylation
**hAd-MSC**	MidPass	−0.0103	<2.2 × 10^−16^	0.000	Hypomethylation
	RS	−0.0202	<2.2 × 10^−16^	0.000	Hypomethylation
	IRR-SIPS	0.0034	<2.2 × 10^−16^	0.000	Hypermethylation
	Dox-SIPS	0.0038	<2.2 × 10^−16^	0.000	Hypermethylation

**Table 2:** Summarization of the sets of differentially methylated positions and genes.

Cell Type	Condition	Number of DMPs	Number of Hypomethylated DMPs	Percentage of Hypomethylated DMPs	Number of Hypermethylated DMPs	Percentage of Hypermethylated DMPs	Number of Unique Genes
**DSF**	MidPass	636	480	75	156	25	366
	RS	25,097	11,880	47	13,217	53	9486
	IRR-SIPS	33	16	48	17	52	25
	Dox-SIPS	31	11	35	20	65	25
**hBM-MSC**	MidPass	64	14	22	50	78	45
	RS	24,707	15,229	62	9478	38	7884
	IRR-SIPS	21	13	62	8	38	16
	Dox-SIPS	63	35	56	28	44	44
**hAd-MSC**	MidPass	1426	1203	84	223	16	829
	RS	25,688	21,592	84	4096	16	7422
	IRR-SIPS	27	11	41	16	59	19
	Dox-SIPS	27	7	26	20	74	23

**Table 3:** Number of KEGG pathways that were enriched in the sets of genes identified with at least one differentially methylated CpG when comparing treated cells with the respective early-passage cells.

		Number of Pathways	
Cell Type	Condition	with Nominal *p*-Value < 0.05	with Adjusted *p*-Value < 0.05
**DSF**	MidPass	17	0
	RS	45	10
	IRR-SIPS	8	0
	Dox-SIPS	1	0
**hBM-MSC**	MidPass	1	0
	RS	33	5
	IRR-SIPS	14	2
	Dox-SIPS	20	4
**hAd-MSC**	MidPass	57	7
	RS	43	8
	IRR-SIPS	11	0
	Dox-SIPS	7	0

**Table 4:** Results of pathway enrichment analysis for DSF after replicative senescence (RS). Only the terms for which adjusted *p*-value reached the statistical significance level of 0.05 are listed. Overlap column indicates ratio between number of genes provided as input list and present in a particular pathway and the total number of genes constituting that pathway. *p*-value and adjusted *p*-value are calculated with Fisher’s exact test. Combined score is computed by taking the logarithm of the *p*-value from Fisher’s exact test and multiplying that by the z-score of the deviation from the expected rank. Rank-based ranking is derived from running the Fisher’s exact test for many random gene sets in order to compute a mean rank and standard deviation from the expected rank for each term in the gene-set library and finally calculating a z-score to assess the deviation from the expected rank.

KEGG Pathway Term in DSF	Overlap	*p*-Value	Adjusted *p*-Value	Combined Score
**RS**				
Glutamatergic synapse	75/114	0.000	0.018	20.928
Adherens junction	49/71	0.000	0.029	21.230
Axon guidance	110/182	0.000	0.029	13.979
Parathyroid hormone synthesis, secretion and action	68/106	0.000	0.030	15.694
cAMP signaling pathway	127/216	0.000	0.031	12.113
Arrhythmogenic right ventricular cardiomyopathy	51/77	0.001	0.033	15.962
Rap1 signaling pathway	123/210	0.001	0.033	11.367
Dopaminergic synapse	81/132	0.001	0.034	12.464
Calcium signaling pathway	138/240	0.001	0.037	10.346
Morphine addiction	58/91	0.001	0.040	13.072

**Table 5:** Results of pathway enrichment analysis for hBM-MSC after replicative, irradiation- and doxorubicin-induced senescence (respectively RS, IRR-SIPS and Dox-SIPS). Only the terms for which adjusted *p*-value reached the statistical significance level of 0.05 are listed. For the description of column names, please refer to Table 4.

KEGG Pathway Term in hBM-MSC	Overlap	*p*-Value	Adjusted *p*-Value	Combined Score
**RS**				
Morphine addiction	54/91	0.000	0.015	20.899
GABAergic synapse	53/89	0.000	0.015	21.046
Axon guidance	96/182	0.000	0.018	14.928
Nicotine addiction	27/40	0.000	0.023	25.946
Synaptic vesicle cycle	46/78	0.000	0.023	17.543
**IRR-SIPS**				
Regulation of actin cytoskeleton	3/218	0.001	0.033	155.910
Melanoma	2/72	0.001	0.038	264.684
**Dox-SIPS**				
Melanoma	4/72	0.000	0.002	318.266
Calcium signaling pathway	5/240	0.000	0.008	92.954
Regulation of actin cytoskeleton	4/218	0.001	0.038	61.131
Leukocyte transendothelial migration	3/114	0.002	0.043	81.200

**Table 6:** Results of pathway enrichment analysis for hAd-MSC after a middle number of passages (MidPass) and replicative senescence (RS). Only the terms for which adjusted *p*-value reached the statistical significance level of 0.05 are listed. For the description of column names, please refer to Table 4.

KEGG Pathway Term in hAd-MSC	Overlap	*p*-Value	Adjusted *p*-Value	Combined Score
**MidPass**				
Glutamatergic synapse	14/114	0.000	0.033	26.984
Phospholipase D signaling pathway	16/148	0.000	0.033	21.973
GnRH signaling pathway	12/93	0.000	0.033	26.654
Thyroid hormone signaling pathway	14/121	0.000	0.033	23.318
Choline metabolism in cancer	12/98	0.001	0.039	23.535
Spinocerebellar ataxia	15/143	0.001	0.040	19.261
Calcium signaling pathway	21/240	0.001	0.041	15.392
**RS**				
ECM-receptor interaction	50/88	0.000	0.022	20.025
Axon guidance	91/182	0.000	0.022	14.152
Nicotine addiction	26/40	0.000	0.022	25.441
Cholinergic synapse	60/113	0.000	0.022	15.193
Morphine addiction	50/91	0.000	0.022	16.266
GABAergic synapse	49/89	0.000	0.022	16.200
Arrhythmogenic right ventricular cardiomyopathy	43/77	0.001	0.028	15.881
Focal adhesion	97/201	0.001	0.030	11.409

**Table 7:** Results of pathway enrichment analysis for genes that were found common for replicative senescence (RS) in human DSF, hBM-MSC and hAd-MSC. Only terms for which adjusted *p*-value reached the statistical significance level of 0.05 are listed. For the description of column names, please refer to Table 4.

KEGG Pathway Term in RS	Overlap	*p*-Value	Adjusted *p*-Value	Combined Score
GABAergic synapse	34/89	0.000	0.000	72.311
Morphine addiction	32/91	0.000	0.000	52.258
Adherens junction	26/71	0.000	0.000	49.409
Cholinergic synapse	35/113	0.000	0.000	37.102
Circadian entrainment	31/97	0.000	0.000	37.029
Calcium signaling pathway	59/240	0.000	0.000	25.074
Dopaminergic synapse	38/132	0.000	0.000	30.938
Glutamatergic synapse	34/114	0.000	0.000	31.755
Nicotine addiction	17/40	0.000	0.000	54.133
Axon guidance	47/182	0.000	0.000	24.963
Arrhythmogenic right ventricular cardiomyopathy	25/77	0.000	0.001	32.301
GnRH secretion	22/64	0.000	0.001	34.784
cAMP signaling pathway	52/216	0.000	0.001	20.612
Rap1 signaling pathway	50/210	0.000	0.001	19.042
Phospholipase D signaling pathway	38/148	0.000	0.002	20.332
Pathways in cancer	104/531	0.000	0.002	13.958
MAPK signaling pathway	64/294	0.000	0.002	15.863
Oxytocin signaling pathway	38/154	0.000	0.004	17.441
Synaptic vesicle cycle	23/78	0.000	0.004	21.827
Adrenergic signaling in cardiomyocytes	37/150	0.000	0.004	17.034
AMPK signaling pathway	31/120	0.000	0.005	17.539
Aldosterone synthesis and secretion	26/98	0.001	0.009	16.726
Parathyroid hormone synthesis secretion and action	27/106	0.001	0.013	14.866
Insulin secretion	23/86	0.001	0.014	15.578
Pentose and glucuronate interconversions	12/34	0.001	0.015	22.599
Bacterial invasion of epithelial cells	21/77	0.001	0.015	15.504
Long-term potentiation	19/67	0.001	0.015	16.346
Ascorbate and aldarate metabolism	11/30	0.001	0.016	23.627
Retrograde endocannabinoid signaling	34/148	0.002	0.018	11.963
Wnt signaling pathway	37/166	0.002	0.019	11.271
Amphetamine addiction	19/69	0.002	0.019	14.792
Focal adhesion	43/201	0.002	0.019	10.593
Bile secretion	23/90	0.002	0.020	13.236
ErbB signaling pathway	22/85	0.002	0.020	13.378
Ras signaling pathway	48/232	0.002	0.021	9.884
Hippo signaling pathway	36/163	0.003	0.022	10.609
PI3K-Akt signaling pathway	68/354	0.003	0.022	8.855
Proteoglycans in cancer	43/205	0.003	0.024	9.680
Non-small cell lung cancer	19/72	0.003	0.026	12.768
Porphyrin and chlorophyll metabolism	13/43	0.004	0.031	14.892
Cocaine addiction	14/49	0.005	0.039	13.156
Growth hormone synthesis secretion and action	27/119	0.006	0.041	9.515
Fc gamma R-mediated phagocytosis	23/97	0.006	0.041	10.017
Protein digestion and absorption	24/103	0.006	0.042	9.672
Estrogen signaling pathway	30/137	0.006	0.042	8.900
Chronic myeloid leukemia	19/76	0.006	0.042	10.541
Gastric cancer	32/149	0.007	0.043	8.596
cGMP-PKG signaling pathway	35/167	0.007	0.044	8.250
T cell receptor signaling pathway	24/104	0.007	0.044	9.314
Chemokine signaling pathway	39/192	0.008	0.048	7.738

## Data Availability

The data presented in this study are openly available in the NCBI Gene Expression Omnibus (GEO) (http://www.ncbi.nlm.nih.gov/geo/ accessed on 28 April 2022) under accession number GSE227160.

## Supplementary Materials

The following supporting information can be downloaded at: https://www.mdpi.com/article/10.3390/cells12060927/s1, Table S1. List of neuro-pathways: KEGG pathways widely related to nerves, nervous system and neurological mechanisms. Table S2. Differentially methylated CpGs with adjusted *p*-value < 0.05 and ∆β > 0.2 in MidPass DSF. Table S3. Differentially methylated CpGs with adjusted *p*-value < 0.05 and ∆β > 0.2 in RS DSF. Table S4. Differentially methylated CpGs with adjusted *p*-value < 0.05 and ∆β > 0.2 in IRR-SIPS DSF. Table S5. Differentially methylated CpGs with adjusted *p*-value < 0.05 and ∆β > 0.2 in Dox-SIPS DSF. Table S6. Differentially methylated CpGs with adjusted *p*-value < 0.05 and ∆β > 0.2 in MidPass hBM-MSC. Table S7. Differentially methylated CpGs with adjusted *p*-value < 0.05 and ∆β > 0.2 in RS hBM-MSC. Table S8. Differentially methylated CpGs with adjusted *p*-value < 0.05 and ∆β > 0.2 in IRR-SIPS hBM-MSC. Table S9. Differentially methylated CpGs with adjusted *p*-value < 0.05 and ∆β > 0.2 in Dox-SIPS hBM-MSC. Table S10. Differentially methylated CpGs with adjusted *p*-value < 0.05 and ∆β > 0.2 in MidPass hAd-MSC. Table S11. Differentially methylated CpGs with adjusted *p*-value < 0.05 and ∆β > 0.2 in RS hAd-MSC. Table S12. Differentially methylated CpGs with adjusted *p*-value < 0.05 and ∆β > 0.2 in IRR-SIPS hAd-MSC. Table S13. Differentially methylated CpGs with adjusted *p*-value < 0.05 and ∆β > 0.2 in Dox-SIPS hAd-MSC. Table S14. Results of pathway enrichment analysis for DSF after a middle number of passages, replicative senescence, irradiation- and doxorubicin-induced senescence. Table S15. Results of pathway enrichment analysis for hBM-MSC after a middle number of passages, replicative senescence, irradiation- and doxorubicin-induced senescence. Table S16. Results of pathway enrichment analysis for hAd-MSC after a middle number of passages, replicative senescence, irradiation- and doxorubicin-induced senescence. Table S17. Summary of DMP sets used in cluster analysis. Figure S1 Gene overlaps among the three types of senescence in (a) DSF, (b) hBM-MSC and (c) hAd-MSC.
